# Validation of the 5-item doctor-patient communication competency instrument for medical students (DPCC-MS) using two years of assessment data

**DOI:** 10.1186/s12909-017-1026-9

**Published:** 2017-10-26

**Authors:** Jean-Sébastien Renaud, Luc Côté

**Affiliations:** 1 Department of Family and Emergency Medicine, Faculty of Medicine, Laval University, Pavillon Ferdinand-Vandry, Bureau 2881-D, (Québec), Québec, G1V 0A6 Canada; 2 Department of Family and Emergency Medicine, Faculty of Medicine, Laval University, Pavillon Ferdinand-Vandry, Bureau 2207-A, (Québec), Québec, G1V 0A6 Canada

**Keywords:** Medical students, Doctor-patient communication, Assessment, Psychometric validation

## Abstract

**Background:**

Medical students on clinical rotations have to be assessed on several competencies at the end of each clinical rotation, pointing to the need for short, reliable, and valid assessment instruments of each competency. Doctor patient communication is a central competency targeted by medical schools however, there are no published short (i.e. less than 10 items), reliable and valid instruments to assess doctor-patient communication competency. The Faculty of Medicine of Laval University recently developed a 5-item Doctor-Patient Communication Competency instrument for Medical Students (DPCC-MS), based on the Patient Centered Clinical Method conceptual framework, which provides a global summative end-of-rotation assessment of doctor-patient communication. We conducted a psychometric validation of this instrument and present validity evidence based on the response process, internal structure and relation to other variables using two years of assessment data.

**Methods:**

We conducted the study in two phases. In phase 1, we drew on 4991 student DPCC-MS assessments (two years). We conducted descriptive statistics, a confirmatory factor analysis (CFA), and tested the correlation between the DPCC-MS and the Multiple Mini Interviews (MMI) scores. In phase 2, eleven clinical teachers assessed the performance of 35 medical students in an objective structured clinical examination station using the DPCC-MS, a 15-item instrument developed by Côté et al. (published in 2001), and a 2-item global assessment. We compared the DPCC-MS to the longer Côté et al. instrument based on internal consistency, coefficient of variation, convergent validity, and inter-rater reliability.

**Results:**

Phase 1: Cronbach’s alpha was acceptable (.75 and .83). Inter-item correlations were positive and the discrimination index was above .30 for all items. CFA supported a unidimensional structure. DPCC-MS and MMI scores were correlated. Phase 2: The DPCC-MS and the Côté et al. instrument had similar internal consistency and convergent validity, but the DPCC-MS had better inter-rater reliability (mean ICC = .61).

**Conclusions:**

The DPCC-MS provides an internally consistent and valid assessment of medical students’ communication with patients.

## Background

Doctor-patient communication is at the heart of medical practice. In Canada, doctor-patient communication is one of the central competencies targeted in medical education programs. In North America, this competency is endorsed not only by the Association of Faculties of Medicine of Canada but also by the Royal College of Physicians and Surgeons of Canada. It is a component of the CANMEDs communicator role [1] and is also a well-established norm in the US [2, 3].

Given the importance of doctor-patient communication in the undergraduate curriculum, it is critical to assess this competency across various situations and with various methods, particularly during clinical rotations where it is possible to observe and evaluate student performance with real patients. However, because doctor-patient communication is just one of several competencies assessed during rotations, there is a need for short, comprehensive, reliable and valid measurement instruments of doctor-patient communication [4]. Furthermore, studies show that there is little gain in measurement precision and score generalizability when using more than five items to assess student clinical performance during rotations [5]. Additionally, our experience at Laval University shows that it is logistically impractical to have 10 or more items on doctor-patient communication alone, when five to seven competencies are being assessed for each clinical rotation completed by the student. To our knowledge, there are no published short (i.e. less than 10 items), reliable and valid instruments to assess doctor-patient communication [6, 7]. For example, a recent systematic review by Zill et al. [8] of available English instruments to assess doctor-patient communication competency found that the shortest instrument had 10 items, nine other instruments had 12 to 20 items, while another 10 instruments had up to 95 items. There is also an instrument published in French [9] that has good content validity and high internal consistency reliability, but its 15 items still make it too long for summative assessments of a range of competencies at the end of- clinical rotations. As a result, different faculties of medicine have often developed their own short instruments whose reliability and validity are either undocumented or unpublished. Finally, as highlighted in a recent systematic review [10], the heterogeneity of the instruments used to assess doctor-patient communication in undergraduate medical education makes it difficult to compare performance across institutions. These realities point to the importance of developing and validating short, but accurate, instruments that assess the communication skills of medical students on clinical rotations.

The Faculty of Medicine of Laval University recently developed a 5-item instrument named the Doctor-Patient Communication Competency for Medical Students (DPCC-MS) to measure doctor-patient communication (Appendix, Table 5). The instrument is based on the Patient Centered Clinical Method (PCCM) conceptual framework suggested by Stewart et al. [11] and provides a global assessment of medical students on doctor-patient communication. Each item is rated on a four-point performance scale: Superior = 4, Expected = 3, Borderline = 2, Insufficient = 1. Raters can also choose the “Not applicable” option, which is coded as a missing value. Items’ scores are averaged into a single synthetic score reflecting overall performance.

The proposed tool has good content validity based on the PCCM framework [11] but excludes two components that are not specifically related to doctor-patient communication competency, namely, “incorporating prevention and health promotion” and “being realistic”. However, because content validity is necessary but not sufficient to support the validity of an instrument [12], further psychometric validation is needed to determine the extent to which the tool is a reliable and valid measure of doctor-patient communication among medical students.

Using the validity framework suggested by the Standards for Educational and Psychological Assessment [12], this study aimed at developing validity evidence for the DPCC-MS based on the response process (inter-rater reliability), internal structure (factor structure, internal consistency, item analysis), and relations to other variables (convergent validity). Specific objectives of the study were:

1. To assess the psychometric properties of the DPCC-MS used at Laval University during clinical rotations;
2. To compare the DPCC-MS psychometric properties to those of a longer scale assessing doctor-patient communication.

## Methods

The study was structured in two phases to meet each of the study objectives.

### Phase 1

The objective of the first phase was to assess the psychometric properties of the DPCC-MS used by Laval University during clinical rotations.

#### Sample and procedures

At Laval University, medical students complete their clinical rotations over a two-year period. Each rotation lasts between three and six weeks, and at the end of each rotation, students receive a summative evaluation based on a standardized evaluation form, which includes DPCC-MS items. For the purposes of this study, we selected all 4991 correctly digitalized assessments from the database of 634 students who completed their clinical rotations between 2011 and 2013. There were 3111 (62%) junior student (i.e. in their first year of clinical rotations) assessments and 1880 (38%) senior student (i.e. in their second year of clinical rotations) assessments.

#### Analyses

We assessed the psychometric properties of the scale by analyzing its internal structure and its relation to other variables, which are two sources of validity evidence [12]. The internal structure of the scale was assessed separately for junior and senior students to see if it was adequate for both groups using internal consistency reliability (i.e. Cronbach’s alpha), inter-item correlations, classical item analysis (i.e. items’ descriptive statistics and discrimination index), and confirmatory factor analysis (CFA). We set .70 as the minimally acceptable value for Cronbach’s alpha [13]. A value between .70 and .79 was deemed acceptable for the instrument, because it is not a single end-of-year or end-of-course assessment in which case a Cronbach’s alpha of at least .80 would have been necessary [14]. Rather, the instrument was used to assess each of the several yearly clinical rotations, each having a different duration. In addition, we calculated the standard error of measurement to estimate the precision of scores [15]. Inter-item correlations were computed to make sure that all items were positively correlated. We also used these correlations to verify if some items were redundant, defined as having a Pearson correlation coefficient of at least .70, meaning that the items shared 50% of variance or more. In conducting the item analysis, we anticipated items means (i.e. difficulty) to be around 3, because it represented the level of performance on the response scale labeled as “Expected”, with most scores lying between 2 and 4. The item discrimination index, which was the corrected item-total correlation, was interpreted as follows: below .20 is poor, between .20 and .29 is modest, between.30 and .39 is good, and .40 or more is very good [13, 16–18]. CFA was estimated using a polychoric correlation matrix and unweighted least squares (ULS) estimation because of the ordinal nature of the response scale [19, 20]. We tested for a unidimensional structure because the instrument was designed to measure a single construct. We interpreted model fit indices following Schermelleh-Engel, Moosbrugger, and Müller’s [21] guidelines for acceptable fit: standardized root mean square residual (SRMR) values less than .10; goodness of fit (GFI) values greater than .90; adjusted goodness of fit (AGFI) values greater than .85; normed fit index (NFI) values greater than .90.

We also assessed the convergent validity of the DPCC-MS by correlating its scores with Multiple Mini Interviews (MMI) scores. The MMI is an admission tool used by the undergraduate medical program to assesses “non-cognitive” abilities, including communication skills [22, 23]. There is typically a two to three-year time lag between the MMI and the beginning of the clinical rotations. We therefore expected a small, yet significant, Pearson correlation coefficient between the scores on the DPCC-MS and those on the MMI. The MMIs were first implemented in 2009, therefore, we could only test its relation with DPCC-MS scores for the sub-sample of 242 students who did their MMI in 2009 or 2010. MMI scores are reported on a standardized scale with a mean of 500 and a standard deviation of 50.

### Phase 2

The objective of the second phase was to compare the psychometric properties of the DPCC-MS to those of a longer scale assessing doctor-patient communication.

#### Sample and procedures

Eleven clinical teachers assessed a total of thirty-five videos using the DPCC-MS and two other instruments: a 13-item doctor-patient relationship skills assessment instrument developed by Côté et al. (published in 2001) (Appendix, Table 6) [9], and a 2-item global assessment of doctor-patient communication skills (see Instruments section). These videos showed the performance of medical students (second year of clinical rotations) at objective structured clinical examination (OSCE) station that assessed doctor-patient communication. Student performance was recorded using a ceiling-mounted camera. Filming students during performance assessments is a fairly standard practice and students knew they would be filmed for a research project, but they did not know at which station. Of the 230 students invited, 167 (73%) volunteered to participate in the study, and we selected the 35 videos in which students were most clearly visible and front facing.

We paired eight of the eleven clinical teacher raters in order to estimate and compare inter-rater reliability for the DPCC-MS and Côté et al. instruments. Each pair of raters assessed five videos, for 20 videos in total (four pairs, each assessing 5 videos). The remaining three raters each assessed five videos, for 15 videos in total (three raters, each assessing five videos).

#### Instruments

Two of the 15-items in the Côté et al. instrument were not used because they didn’t apply to the clinical scenario of the OSCE station. Those two items were: “Asks the patient to describe how his/her health problems are affecting his/her daily life” and “Asks the patient to express his/her perception of his/her symptoms”. Each of the remaining 13 items were measured on a 4-point response scale: 1 = Completely disagree, 2 = Somewhat disagree, 3 = Somewhat agree, 4 = Completely agree. The mean total score on this instrument could vary between 1 and 4, with a higher score meaning better skills. For the global assessment of doctor-patient communication skills, the first item was: “If you had to give this student a score for his/her communication with patient skills, where 1=Insufficient and 10=Superior, what would it be?” The second item was: “Globally assess the candidate's ability to communicate effectively with the patient and establish a good relationship with him/her.” For this item, performance was rated on a 4-point scale running from 1 = poor to 4 = excellent, and where 3 was labeled as “adequate performance”. Adequate performance was defined to raters as being able to “put the patient at ease during the interview using both verbal and non-verbal communication; addresses the patient with respect, tact, and delicacy; interested in the patient's needs and adapts to the different needs while taking into account the tasks he/she has to perform”. These two global assessment items were summed up to compute a global assessment score that ranged from 2 to 14, with a higher score meaning better skills.

#### Analyses

The analyses focused on three types of validity evidences, those based on the internal structure, on the relation to other variables, and on the response process. More precisely, we compared the psychometric properties of the DPCC-MS and the Côté et al. scales based on their internal consistency, standard error of measurement, coefficient of variation, convergent validity, and inter-rater reliability. Because 20 of the 35 videos were assessed twice, that is by a pair of raters rather than by a single rater, we randomly selected a single rating for each of these videos to avoid dependent observations. We therefore had a database of 35 different videos, each assessed by one of the eleven raters using three instruments. Using this database, we estimated the internal consistency reliability using Cronbach’s alpha for each instrument, and conducted descriptive statistics for the total scores. The coefficient of variation, a relative measure of dispersion, was estimated in addition to the standard deviation because the total scores on the three instruments were expressed on different scales. Convergent validity of the DPCC-MS and the Côté et al. instrument was assessed by estimating their correlation (Pearson’s coefficient) with the global assessment score. To test if the difference between these two correlation coefficients was statistically significant at an alpha level of .05, we checked if their 95% confidence intervals were overlapping. Convergent validity of the DPCC-MS was also assessed by estimating its correlation with the Côté et al. scale. Finally, for the 20 videos that were assessed by pairs of raters, we estimated the inter-rater reliability of both the DPCC-MS and Côté et al. scale for all pairs of raters using the intraclass correlation coefficient (ICC). More precisely, we estimated the reliability of the ratings of a single rater using a two-way mixed, single-measures, consistency ICC [24]. We used Cicchetti’s [25] guidelines to interpret ICC values: below .40 is poor, between .40 and .59 is fair, between .60 and .74 is good, and between .75 and 1.00 is excellent.

## Results

### Phase 1

#### Descriptive statistics, inter-item correlations, internal consistency, and standard error of measurement

The mean score for junior students (M = 3.27, SD = .30, Min. = 2.60, Max. = 4.00) was higher than for senior students (M = 3.18, SD = .29, Min. = 2.80, Max. = 4.00), as indicated by a statistically significant Kruskall-Wallis non-parametric one-way analysis of variance (χ^2^(1) = 211.37, *p* < .0001). Inter-item correlations ranged from .26 to .52 for junior students and from .39 to .66 for senior students. All inter-item correlations were positive and statistically significant at the .01 level, and none were above .70, indicating that there were no redundant items. Cronbach’s alpha values, over .70, were acceptable. Cronbach’s alpha was higher for senior (α = .83) than for junior students (α = .75), and the Hakstian and Whalen [26] test showed that the difference is statistically significant, χ^2^(1) = 66.35, *p* < .0001. This higher Cronbach’s alpha value, combined with almost the same standard deviation as junior students, resulted in a smaller standard error of measurement for senior students (.12 vs. .15).

#### Item analysis

We conducted an item analysis on the data for junior and senior medical students (Table 1) and the results show that all items have either good (≥ .30) or very good (≥ .40) discrimination. Item means were all near 3, corresponding to an “Expected” level of performance. Item 1 was the easiest (highest mean) and the least discriminating (discrimination index between .39 and .53) in both groups, and item 2 was highly discriminating in both groups.

**Table 1:** Item analysis for junior and senior students

Students	Item	Discrimination	Mean	Std	Min.	Max.
Junior	1	.39	3.56	.50	2.00	4.00
	2	.61	3.19	.40	3.00	4.00
	3	.53	3.27	.45	2.00	4.00
	4	.54	3.15	.36	3.00	4.00
	5	.58	3.19	.39	2.00	4.00
Senior	1	.53	3.34	.47	2.00	4.00
	2	.68	3.13	.34	2.00	4.00
	3	.68	3.16	.37	2.00	4.00
	4	.62	3.14	.35	3.00	4.00
	5	.67	3.14	.35	3.00	4.00

*Std* standard deviation, *Min* minimum, *Max* maximum

#### Confirmatory factor analyses

We conducted separate confirmatory factor analyses on the junior and senior medical student data (Table 2). These analyses show that a unidimensional factor structure provided a good fit to the data: GFI, AGFI, and NFI were ≥ .99, and SRMR was ≤ .03. Item loadings on the single factor were strong: between .66 and .90 on the junior student dataset and between .81 and .92 on the senior student dataset. The single factor explained between 44% and 81% of items variance for junior students and between 65% and 85% of items variance for senior students.

**Table 2:** Results of the confirmatory factor analysis

Indice		Junior	Senior
Fit indices	SRMR	.030	.024
	GFI	.998	.999
	AGFI	.994	.996
	NFI	.997	.999
Item loadings	Item 1	.66	.81
	Item 2	.90	.92
	Item 3	.77	.92
	Item 4	.83	.87
	Item 5	.86	.92
R^2^	Item 1	.44	.65
	Item 2	.81	.85
	Item 3	.60	.84
	Item 4	.68	.75
	Item 5	.75	.85

*SRMR* standardized root mean square residual, *GFI* goodness of fit, *AGFI* adjusted goodness of fit, *NFI* normed fit index, *R* ^*2*^ proportion of variance in item accounted for by the common factor

#### Convergent validity: Correlation with MMI scores

We tested the linear relation between DPCC-MS scores and admission MMI scores. MMI scores for the sub-sample used in this analysis (*n* = 242) ranged from 431.74 to 629.063 (*M* = 530.70, *SD* = 35.24). The Pearson correlation coefficient between DPCC-MS mean scores and MMI scores was .22 (*p* = .001).

### Phase 2

#### Descriptive statistics and reliability

Cronbach’s alpha coefficient, standard error of measurement, and descriptive statistics for the DPCC-MS, the Côté et al. instrument, and the global rating scale are presented in Table 3. Cronbach’s alpha coefficient for all three scales is good, and both the DPCC-MS and the Côté et al. instrument have a similar high internal consistency, above .90. The coefficient of variation shows that scores on the DPCC-MS exhibit more variability that those on Côté et al. scale, resulting in a smaller standard error of measurement for the latter.

**Table 3:** Cronbach’s alpha and descriptive statistics for the DPCC-MS, Côté et al., and global rating instruments

	Cronbach’s alpha	SEM	Mean	SD	C.V.	Min.	Max.
DPCC-MS	.91	.23	2.81	.76	27%	1.20	4.00
Côté et al.	.94	.14	3.40	.58	17%	2.08	4.00
Global	.88	.97	9.74	2.79	29%	3.00	14.00

*SEM* standard error of measurement, *SD* standard deviation, *C.V.* coefficient of variation, *Min* minimum, *Max.* maximum

#### Convergent validity

We estimated the Pearson correlation coefficient between the DPCC-MS and the global assessment scale. The measures were highly correlated *r* = .90 (*p* < .0001), 95% CI [.81, .95]. We also estimated the Pearson correlation coefficient between the Côté et al. scale and the global assessment scale, which were highly correlated, *r* = .89 (*p* < .0001), 95% CI [.78, .94]. Comparing the 95% confidence intervals shows that the strength of the correlation between these two scales and the global assessment scale is not statistically different. Finally, we estimated the correlation between the scores on the DPCC-MS and those on the Côté et al. scale. The Pearson correlation coefficient was .87, *p* < .0001, 95% CI [.76, .93].

#### Inter-rater reliability

We estimated the inter-rater reliability of both the DPCC-MS and the Côté et al. scale for all four pairs of raters using a two-way mixed, single-measures, consistency ICC (Table 4). Using Cicchetti’s [25] guidelines, the DPCC-MS’s inter-rater reliability is fair to excellent for three of the four raters pairs, and poor for one pair. Côté et al. scale’s inter-rater reliability is fair to excellent for two of the four rater pairs, and poor for the two others. Overall, the DPCC-MS resulted in higher levels of inter-rater reliability than the Côté et al. scale, which were good and fair respectively based on the mean and median ICC for the four rater pairs. Rank ordering the pairs of raters based on their inter-rater reliability resulted in the same ranking irrespective of the scale used to assess the videos: pair 2, pair 1, pair 3, and pair 4 (descending order).

**Table 4:** Intraclass correlation coefficient (single measures) for the DPCC-MS and the Côté et al. instrument

Raters pair	DPCC-MS		Côté et al.	
	ICC	Interpretation	ICC	Interpretation
1	.71	Good	.59	Fair
2	.82	Excellent	.84	Excellent
3	.54	Fair	.32	Poor
4	.35	Poor	.03	Poor
Mean ICC	.61	Good	.45	Fair
Median ICC	.62	Good	.46	Fair

*ICC* two = way, single-measures, consistency intraclass correlation coefficient

## Discussion

The purpose of this study was to assess the validity of the DPCC-MS, a short 5-item instrument to assess doctor-patient communication competency at the end of clinical rotations. We assessed validity based on the instrument’s response process, internal structure and relation to other variables using two years of assessment data. In the first phase of the study, we estimated the psychometric properties (i.e. item difficulty and discrimination, dimensionality, internal consistency reliability, and convergent validity) of the instrument using existing clinical rotation assessment data. In the second phase, we compared the psychometric properties (i.e. internal consistency reliability, coefficient of variation, convergent validity, and inter-rater reliability) of the instrument to those of the longer Côté et al. scale, which has good psychometric properties. Results show that the DPCC-MS is a short unidimensional instrument that provides an internally consistent and valid assessment of medical student doctor-patient communication, but that inter-rater reliability can differ significantly between rater pairs. Its psychometric properties were similar to the Côté et al. instrument.

The DPCC-MS has good psychometric properties for both junior and senior medical students. However, its internal consistency reliability is significantly lower for junior students than for senior students (.75 versus .83). One explanation for this finding may be that performance is assessed against an expected level of performance (i.e. superior/expected/borderline/insufficient), but assessors have clearer and more uniform performance expectations for students at the end of MD training (senior students) than for those who are beginning their clinical rotations (junior students), meaning that their judgment is more reliable for senior students on clinical rotations than for their junior peers. Another explanation could be that assessors are more lenient with junior students on rotation, and rate a wider range of performance as acceptable (i.e. “expected” and “superior” categories of the rating scale). This would result in a less precise assessment of student performance and more measurement error. It would also explain why senior students have a lower mean score.

For both junior and senior students on rotations, items 1 “Establishes a good relationship with the patient using the patient-centered clinical method” and 3 “Understands the patient as a whole person (in psycho-social and cultural context) during the interview” were the easiest (i.e. highest mean scores). Item 4 “Checks that the patient has a good understanding of his/her problem” was the hardest (i.e. lowest mean score) for junior students and among the hardest items for senior students. This suggests that early in their clinical rotations, especially during their first year, students focus more on fostering a good relationship with the patient and on trying to grasp the clinical portrait than on trying to explore the patient’s perception of his/her problem. The item discrimination index shows that item 1, which has the mean score the closest to the maximum possible score, is the least discriminating. It is well established that very easy (or very hard) items have a tendency to have lower discriminatory power [27]. In addition, assessors might have paid more attention to the first half of the sentence “Establishes a good relationship with the patient”, a task that is relatively easy to achieve and does not discriminate much between students, than on the second half of the sentence “using the patient-centered clinical method”, which is harder to put into practice and where there is more variation among students. Studying the cognitive response process of assessors, for example using cognitive interviews [28], would help to understand why item 1 is easier and less discriminating. For both junior and senior students on clinical rotations, items 2 “Explores the emotional experience of the patient in line with the patient-centered clinical method” and 5 “Uses appropriate attitudes and strategies in the therapeutic relationship with the patient (respect, empathy, etc.)” are among the most discriminating items. This finding makes sense from a theoretical perspective because these two items represent the core of the PCCM.

We found no differences in the internal consistency reliability and convergent validity of the DPCC-MS and the longer Côté et al. scale. This supports the use of the DPCC-MS as a quick and reliable doctor-patient communication assessment tool for students. It also echoes the results of Kreiter et al. [5], who found that there is little gain in reliability when using more than five items to assess clinical performance of medical students. Nevertheless, longer instruments like the Côté et al. scale might have better content validity. Moreover, the DPCC-MS is more suitable for summative than for formative assessment. A longer, more detailed scale, is better for giving specific feedback because it helps pin-point the students’ strengths and weaknesses [29]. The fact that the DPCC-MS has a comparable level of internal consistency reliability seems counter-intuitive because Cronbach’s alpha has a tendency to increase as the number of items increase [30]. We explain this, at least in part, by the fact that DPCC-MS’s scores are more variable as indicated by the coefficient of variation, meaning its items may be more sensitive to individual differences.

Furthermore, our results suggest that the DPCC-MS has, on average, good inter-rater reliability, compared to a fair inter-rater reliability for Côté et al. scale. However, we observed relatively large differences in inter-rater reliability between rater pairs, with two having only poor to fair reliability, and the other two having good to excellent reliability. This is a concern particularly in the context of summative assessment. Many factors can affect inter-rater reliability, some related to the assessment instrument (e.g. problems with the instructions or the items), the raters (e.g. training, experience), the level of standardization of the assessment setting, etc. Factors relating to the DPCC-MS might explain these results, but the fact that two pairs of raters reached good to excellent inter-rater reliability leads us to hypothesize that factors related to the raters and the study were more important. For instance, we did not train the raters, which may have resulted in higher variation in observed scores. Furthermore, it is possible that some pairs were naturally more consistent in the way they assessed doctor-patient communication than others. Moreover, the fact that raters had to rate student performance on video could have lowered inter-rater reliability for some rater pairs, as non-verbal communication might have been harder to observe and assess indirectly. For example, facial expressions and eye movements, when filmed using ceiling mounted cameras, may not be as easy to observe as through direct live observation. In sum, the results concerning the DPCC-MS’s inter-rater reliability are inconsistent. Even though good to excellent levels of inter-rater reliability are possible for some pairs of raters, the variation in inter-rater reliabilities suggests that multiple ratings might be necessary to obtain a reliable measure of performance. Further investigation is needed to learn what factors affect the DPCC-MS’s inter-rater reliability.

There are some limits to this study. First, the instrument was only tested in its French version at Laval University, a French-speaking faculty of medicine. Given that the psychometric properties of a measurement instrument are affected by the context of its use, we suppose that different results could be obtained at another medical school and in another language. Second, the second phase of our study used a small sub-sample of videos, which limits the generalizability of our results. This limited sample also limits the precision of our ICC estimates. Lastly, other validity evidences of the DPCC-MS will need to be documented. For instance, because this tool is designed for summative assessments, it will be important to test for differential item functioning to ensure there is no item bias toward any specific group (e.g. a specific ethnic or gender group).

### Practice implications

The DPCC-MS has half the number of items of the shortest instrument reported by Zill et al. [8] in their systematic review. Our results suggest that it is nevertheless possible to make a reliable and valid assessment of medical student doctor-patient communication competency using this short 5-item instrument. This is important for medical educators because several competencies need to be assessed at the end of each clinical rotation, requiring that each be measured with a limited number of items. The DPCC-MS could be used by other medical faculties in the assessment of medical students as long as they ensure that it produces reliable and valid results in their context. Other medical faculties could also adapt the DPCC-MS to better suit their needs, or to jump start the development of their own short instrument using the work presented here. In sum, we believe that this study, and the DPCC-MS, will help medical educators make short, reliable and valid global assessments of the doctor-patient communication competency of medical students. However, it should be remembered that short and single assessment instruments alone cannot capture the full complexity of a competency [31].

## Conclusions

Medical students on clinical rotations have to be assessed on several competencies at the end of each clinical rotation, pointing to the need for short, reliable, and valid assessment instruments of each competency. This study assessed the validity of the DPCC-MS instrument, a short 5-item scale intended to assess doctor-patient communication competency at the end of clinical rotations. The DPCC-MS appears to be a unidimensional instrument that provides an internally consistent and valid assessment of students’ doctor-patient communication. Its psychometric properties are similar to those of a longer, validated scale. However, further attention should be given to improving inter-rater reliability. In addition, there is a need to test the DPCC-MS in other medical schools, document other validity evidences, such as the cognitive response process of the assessors, and test for differential item functioning.

## Appendix

**Table 5:** The DPCC-MS

Give your assessment of this student’s performance on each item using the provided rating scale, where “Expected” corresponds to the usual performance of a student at this academic level.		Superior	Expected	Borderline	Insufficient	Not applicable
1	Establishes a good relationship with the patient using the patient-centered clinical method	4	3	2	1	N/A
2	Explores the emotional experience of the patient in line with the patient-centered clinical method	4	3	2	1	N/A
3	Understands the patient as a whole person (in psycho-social and cultural context) during the interview	4	3	2	1	N/A
4	Checks that the patient has a good understanding of his/her problem	4	3	2	1	N/A
5	Uses appropriate attitudes and strategies in the therapeutic relationship with the patient (respect, empathy, etc.)	4	3	2	1	N/A

**Table 6:** Côté et al. (2001) doctor-patient relationship skills assessment instrument

For each item, circle the number corresponding to your opinion.		Completely Disagree	Somewhat Disagree	Somewhat Agree	Completely Agree
1	Asks patient to describe how his/her health problems are affecting his/her daily life	1	2	3	4
2	Asks patient to give his/her perception of his/her symptoms	1	2	3	4
3	Asks patient to express his/her concerns about his/her symptoms	1	2	3	4
4	Asks patient for his/her expectations of the visit	1	2	3	4
5	Takes the patient’s opinion and concerns into account throughout the interview	1	2	3	4
6	Gives patient time to express himself/herself, and when it is necessary to interrupt the patient, does so in a tactful manner.	1	2	3	4
7	Uses open-ended and closed-ended questions appropriately	1	2	3	4
8	Responds appropriately to patient’s non-verbal communication	1	2	3	4
9	Avoids being aloof and abrupt with patient	1	2	3	4
10	Respects patient’s opinions	1	2	3	4
11	Looks at patient when speaking to patient and when patient is speaking	1	2	3	4
12	Expresses himself/herself clearly and precisely	1	2	3	4
13	Explains the proposed course of action	1	2	3	4
14	Avoids medical jargon	1	2	3	4
15	Checks throughout the interview to ensure that the patient understands	1	2	3	4

## Abbreviations

AGFI: Adjusted goodness of fit
CFA: Confirmatory factor analysis
CI: Confidence interval
DPCC-MS: Doctor-patient communication competency for medical students
GFI: Goodness of fit
ICC: Intraclass correlation coefficient
M: Mean
Max: Maximum
Min: Minimum
MMI: Multiple mini interviews
NFI: Normed fit index
OSCE: Objective structured clinical examination
PCCM: Patient centered clinical method
SD: Standard deviation
SRMR: Standardized root mean square residual
ULS: unweighted least squares
